# Supplementary material for: Functional Role of Single-Nucleotide Polymorphisms on IFNG and IFNGR1 in Humans with Cardiovascular Disease
Source: Int J Mol Sci. 2025 Sep 10;26(18):8806. doi: 10.3390/ijms26188806 (PMC12865476; doi:10.3390/ijms26188806)
Supplement: Supplementary file 1 [file ijms-26-08806-s001.zip › supp_figures_all.pdf]

SUPPLEMENTAL FIGURES:

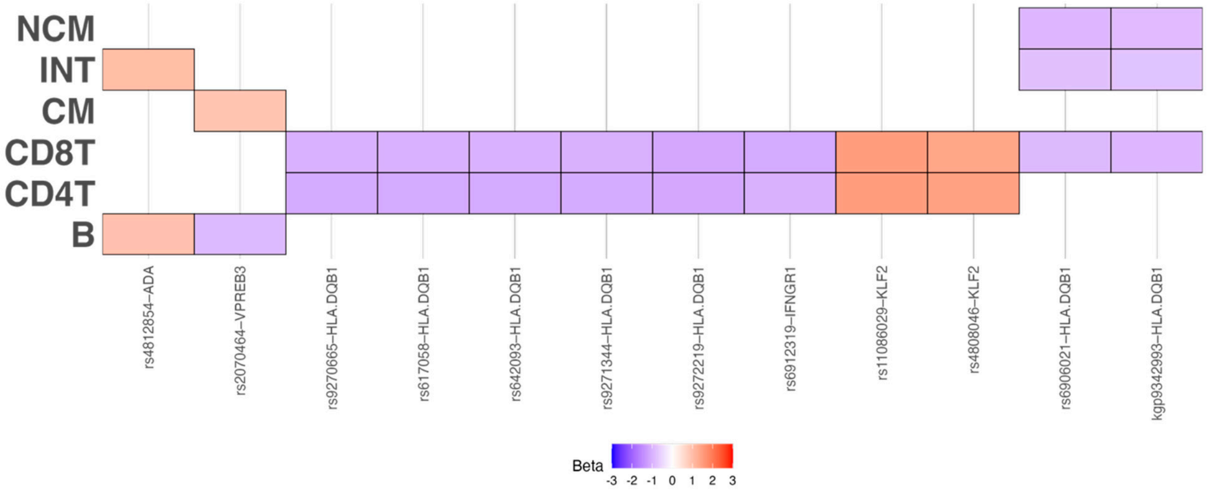

Supplemental Figure S1: All eQTLs that were significant in multiple cell types. 9 eQTLs were significant in 2 cell types and 3 in more than 2 cell types.

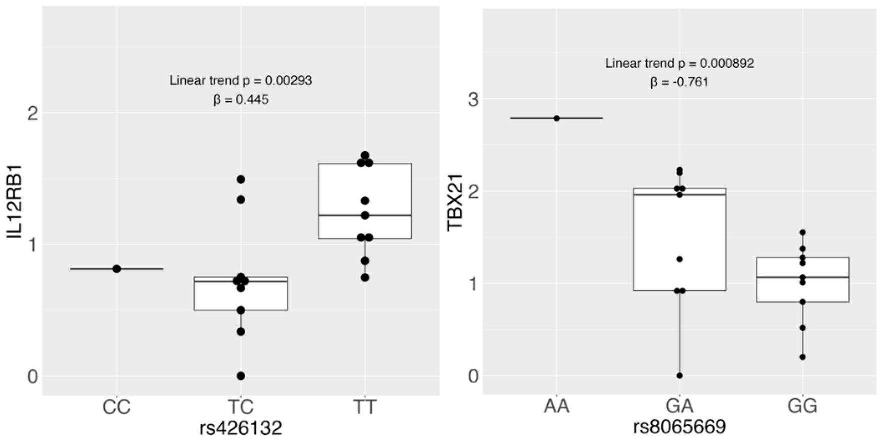

(a) **(b)**

Supplemental Figure S2: Additional CD4+ T cell sc-eQTLs impacting eGenes related to T helper cell differentiation. **(a)** Effect of one sc-eQTL on IL12RB1 expression in CD4+ T cells; **(b)** Effect of one sc-eQTL on TBX21 expression in CD4+ T cells.

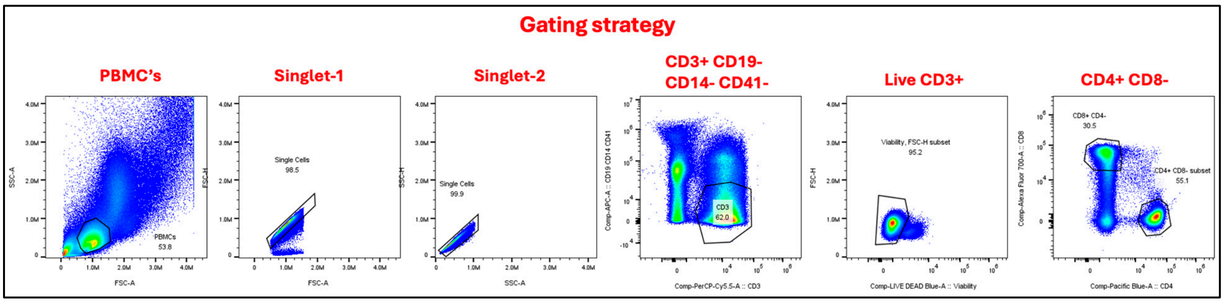

Supplemental Figure S3: Gating strategy for isolating CD4+ T cells during flow cytometry studies

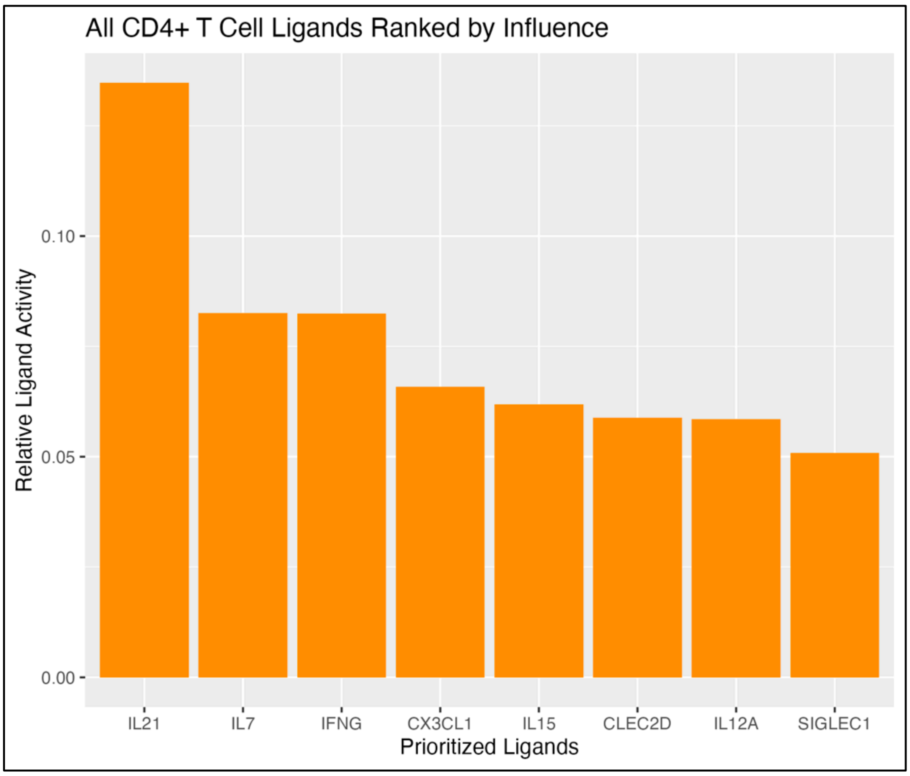

(a)

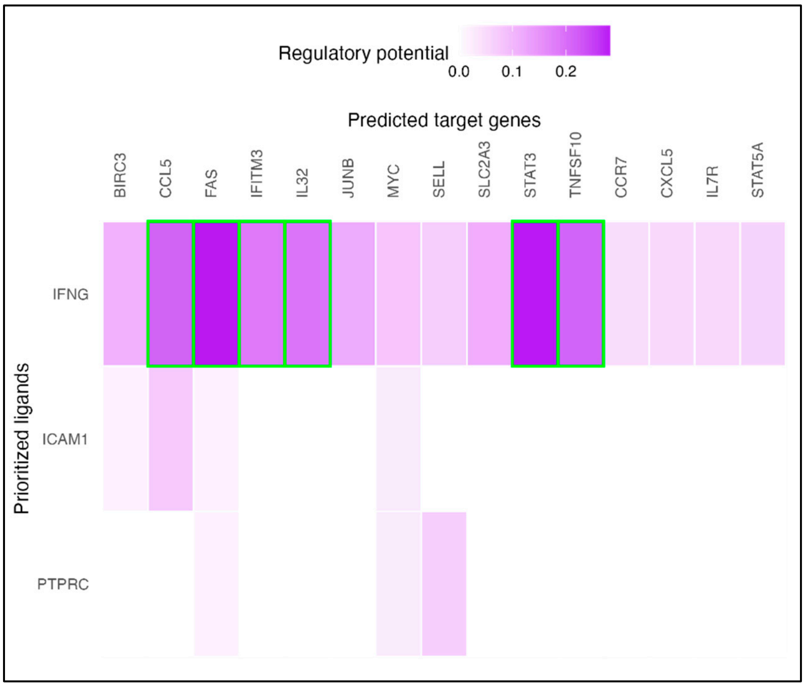

(b)

Supplemental Figure S4: (a) NicheNet analysis ranking the significance of all possible ligands to CD4+ T cells by their influence on downstream gene expression. IFNy (IFNG) is the 3rd most significant signal; **(b)** Top genes influenced by IFNG, ICAM1, and PTPRC reception. Outlined in green are all genes included in the co-expression score to identify IFNG-influenced CD4+ T cells.
